# Signal-Decay Based Approach for Visualization of Buried Defects in 3-D Printed Ceramic Components Imaged with Help of Optical Coherence Tomography

**DOI:** 10.3390/ma16103607

**Published:** 2023-05-09

**Authors:** Malgorzata Kopycinska-Müller, Luise Schreiber, Eric Schwarzer-Fischer, Anne Günther, Conner Phillips, Tassilo Moritz, Jörg Opitz, Yeong-Jin Choi, Hui-suk Yun

**Affiliations:** 1 Fraunhofer Institute for Ceramic Technologies and Systems (IKTS), 01277 Dresden, Germany; 2 Korea Institute of Materials Science (KIMS), Changwon-si 51508, Republic of Korea

**Keywords:** optical coherence tomography, quality control, defect detection, 3-D printing, image processing, image analysis, sintering, ceramic

## Abstract

We propose the use of Optical Coherence Tomography (OCT) as a tool for the quality control of 3-D-printed ceramics. Test samples with premeditated defects, namely single- and two-component samples of zirconia, titania, and titanium suboxides, were printed by stereolithography-based DLP (Digital Light Processing) processes. The OCT tomograms obtained on the green samples showed the capability of the method to visualize variations in the layered structure of the samples as well as the presence of cracks and inclusions at depths up to 130 µm, as validated by SEM images. The structural information was visible in cross-sectional images as well as in plan-view images. The optical signal measured from the printed zirconia oxide and titanium oxide samples showed strong attenuation with depth and could be fit with an exponential decay curve. The variations of the decay parameter correlated very well with the presence of defects and material variation. When used as an imaging quantity, the decay parameter projects the position of the defects into 2-D (X,Y) coordinates. This procedure can be used in real time, it reduces the data volume up to 1000 times, and allows for faster subsequent data analysis and transfer. Tomograms were also obtained on sintered samples. The results showed that the method can detect changes in the optical properties of the green ceramics caused by sintering. Specifically, the zirconium oxide samples became more transparent to the light used, whereas the titanium suboxide samples became entirely opaque. In addition, the optical response of the sintered zirconium oxide showed variations within the imaged volume, indicating material density variations. The results presented in this study show that OCT provides sufficient structural information on 3-D-printed ceramics and can be used as an in-line tool for quality control.

## 1. Introduction

Advanced ceramics are incredible materials that can be designed to achieve combinations of desired primary properties such as electrical conductivity and biocompatibility, as well as extremely beneficial secondary properties such as enhanced toughness, heat and corrosion resistance, or wear endurance. Due to the wide range of achievable properties, advanced ceramics find purpose in many high-performance applications in the fields of biomedicine, defense, energy, and aerospace. These fields demand strict safety requirements in order to maintain the safety of patients, users, and bystanders. As such, advanced ceramics must be reliable, free of relevant defects, and of the highest quality [1].

Ceramic components must often be formed into complex shapes to meet the design requirements of the encompassing system. The shaping of ceramics can take several different paths, including powder injection molding, tape casting, slip casting, extrusion, and many others. The shaping method selected is dependent on the material itself, complexity of the required form, and the application. However, none of the traditional shaping methods can match the form-design flexibility of additive manufacturing (AM) methods.

The first implementation of AM technologies for the forming of ceramic components took place over 30 years ago [2,3]. A historical overview on the additive manufacturing of structural ceramics has been published recently by Pelz et al. [4]. The introduction of the ceramics into AM technologies occurred about 10 years later than metal and plastic. Since then, however, ceramic structures have been printed by all seven families of AM technologies: Vat Photopolymerization [5], Powder Bed Fusion [6], Binder Jetting [3], Material Jetting [7], Sheet Lamination [8], Material Extrusion [9], and Directed Energy Deposition [10]. Continuous progress is being made to realize dense and mechanically stable printed ceramic structures. Depending on the process, the dimensions of the printed part can vary from micrometers to centimeters. One must keep in mind that 3-D ceramic printing is an indirect printing process, as the printed ceramic parts must be de-bound and sintered to achieve the desired material properties [11]. During these processes, shrinkage of the printed part is a common and undesired side effect and must be accounted for in the design.

The mechanical robustness of ceramics is contingent upon the strict absence of defects. Printing dense and defect-free complex ceramic structures is challenging regardless of the technology selected for the task [12,13]. As the additive manufacturing of ceramics is such a complex process, involving a multitude of parameters that may influence the final quality of the sintered product, it is crucial that monitoring systems are integrated into the process.

**Figure 1:**
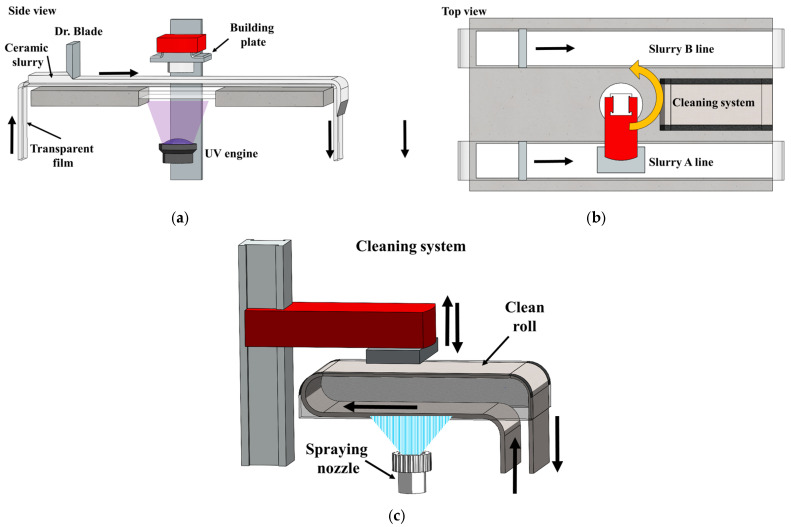
Schematic view of the Multi-CAMP manufacturing process developed by KIMS. side view (**a**), top view (**b**), and cleaning system (**c**).

We intended to combine an electrically insulating, but mechanically strong, ceramic component with an electrically conductive ceramic material. It was decided that both materials should be co-processed by lithography-based additive manufacturing for increasing the geometrical freedom in the design of the manufactured component. Two lithography-based AM systems have been selected to print the test objects, namely a commercially available Lithoz printer [14] and the multi-ceramic additive manufacturing process (Multi-CAMP). The Multi-CAMP process has been developed at the Korea Institute of Materials Science (KIMS) [15,16]. The Multi-CAMP technology (Figure 1a) uses the bottom-up approach of the lithography-based AM process where a whole layer of a light-curable suspension is exposed at once from below through a transparent carrier band to the light source consisting of a digital light module. The Multi-CAMP device has two carrier bands (A line and B line) on which different light-curable suspensions may be applied by different extrusion nozzles (Figure 1b). The extruded suspensions are smoothed into thin films by a doctor blade. Once the layer of material “A” is exposed to the light and solidified, the sample holder can be moved via rotation to the cleaning platform, which is located between the two carrier bands. The cleaning system in Multi-CAMP utilizes a spraying nozzle and cleaning roll, soaked with washing liquid, to remove any remaining ceramic slurry (as shown in Figure 1c). The printed element is “stamped” several times on the cleaning roll to remove any residual ceramic slurry. Once the element is cleaned, it is moved to carrier band B, where the second ceramic slurry is extruded, smoothed, and exposed to the light. The cleaning step is then repeated on a continuously replaced cleaning roll. This process is repeated until the green ceramic part is completed. Next, the green part is removed from the building platform and must be de-bound and sintered to attain the final ceramic properties.

For attaining the desired combination of electrical conductivity in one material and electrical insulation plus mechanical strength in the other, we initially chose titanium suboxides with the symbolic formula Ti_x_O_2x−1_ (which covers a mixture of many suboxides) as the electrical conductors and ZrO_2_ as the insulator. However, titanium suboxides are not translucent for visible light—they are darkly colored and have a high absorption. For that reason, they cannot be used directly for lithography-based AM processes. Instead, we selected titania as it is translucent to wavelengths longer than 400 nm and combined it with zirconia in the Multi-CAMP process for attaining complex-shaped green parts. During sintering under a reducing hydrogen-containing atmosphere, the titania is transformed into titania suboxide by removing a certain content of oxygen from the lattice. After sintering, the titanium suboxide was opaque and electrically conductive, maintaining the original specification. Unfortunately, during the chemical transition from titania to titanium suboxide, the material loses its mechanical strength, which must be compensated for by the densified zirconia compound partner.

The ideal quality control system for 3-D printing processes should be fast (as to not interfere with the printing process), contactless, robust, and capable of the evaluation of sample geometry and topography. The ability of providing volumetric information from at least one printing layer and the interface with the underlaying layer is invaluable. There are different approaches to quality control in the field of additive manufacturing. A review by Kim et al. [17] reports on quality improvement in 3-D-printed structures resulting from quality control research. The research concentrated on a detailed analysis of the printed product and the printing parameters used. Another review by Lee [18] summarizes the quality control methods in metal additive manufacturing via numerical methods; monitoring possibilities including those of thermal imaging, optical imaging, spectroscopy, and acoustic methods are evaluated as well. Considering the character of additive manufacturing of metals, thermal information on the melt-pool is especially important for assuring the quality of the product. Computer tomography methods are also mentioned, but those are limited to ex-situ applications.

Laser scanning methods for quantitative surface imaging are also of importance for additive manufacturing as they provide the information on the resulting geometry and surface quality of the printed product. Examples of the application of 3-D desktop scanners for the quality evaluation of various 3-D printing technologies were presented in [19,20]. In those studies, the laser scanning approach was applied ex situ. Commercially available systems with built-in laser scanners for topography evaluation can be found.

OCT is a contactless and fast tomographic imaging method, and as such it has a large potential as in-line monitoring system. A prior report on the in-situ OCT observation of metal powder beds, as well as the 3-D printing of metal and bio structures, can be found in [21]. Preliminary OCT results on 3-D-printed ceramics showing the potential of the method for in-line monitoring have been shown in [22]. A recent paper by Zorin et al. [23] describes a next-generation OCT system operating at wavelengths around 3.6 µm, enabling greater penetration depths in ceramic materials, with special focus on 3-D-printed ceramics. This development is of note as it shows that OCT systems can be improved and/or custom designed to fit the specific application of imaging ceramics and other turbid materials.

In this report, we too have demonstrated the potential of Optical Coherence Tomography as a versatile quality control tool for 3-D printing processes of ceramics. In the following sections, we will describe the samples investigated, as well as the physical principle of the method. We will present examples of images extracted from the tomograms, showing the presence of defects such as pores, cracks, and cross-contamination. We will also explain our approach for data analysis, which allows for data reduction, faster data transfer, and simplified visualization.

## 2. Materials and Methods

The samples were printed by two different printers, namely a lithography-based commercially available Lithoz printer and the Multi-CAMP system developed at KIMS [14,15]. Details of 3-D printing zirconia and titanium suboxides with the Lithoz printer can be found in [14]. A light of wavelength 405 nm and an intensity of 10 mW/cm^2^ was used to cure TiO_2_ and ZrO_2_ slurries in the Multi-CAMP system. As the two materials have different light curing demands, the curing times were 1 s and 2.5 s for TiO_2_ and ZrO_2_, respectively. The thickness of each laminated layer was 20 µm. The cleaning procedure (see previous section) demanded seven stampings to remove any remains of uncured material and prevent cross-contamination.

The samples were single- and two-component ceramics. The printing parameters were selected to purposefully create defects to test the ability of the OCT method to detect them. The defects of interest were pores, cracks, delamination, and cross-contamination. OCT measurements were performed on samples in green and sintered states. The samples are listed in Table 1.

Three blank samples were printed from different powders in the Lithoz printer. The TiO_2_ slurries were prepared from KRONOS 1002 powders from Kronos International, Inc., Leverkusen, Germany, and CORTIOX, Cosmochem in Korea. The mean grain sizes were 0.4 µm and 0.3 µm for KRONOS and CORTIOX powders, respectively. The ZrO_2_ samples were printed from TZ-3YS-E powder partially stabilized with 3 mol.-% yttria. The powder was supplied by Tosoh Bioscience A.G, Griesheim, Germany. The mean grain size of the ZrO_2_ powder was 0.7 µm.

**Figure 2:**
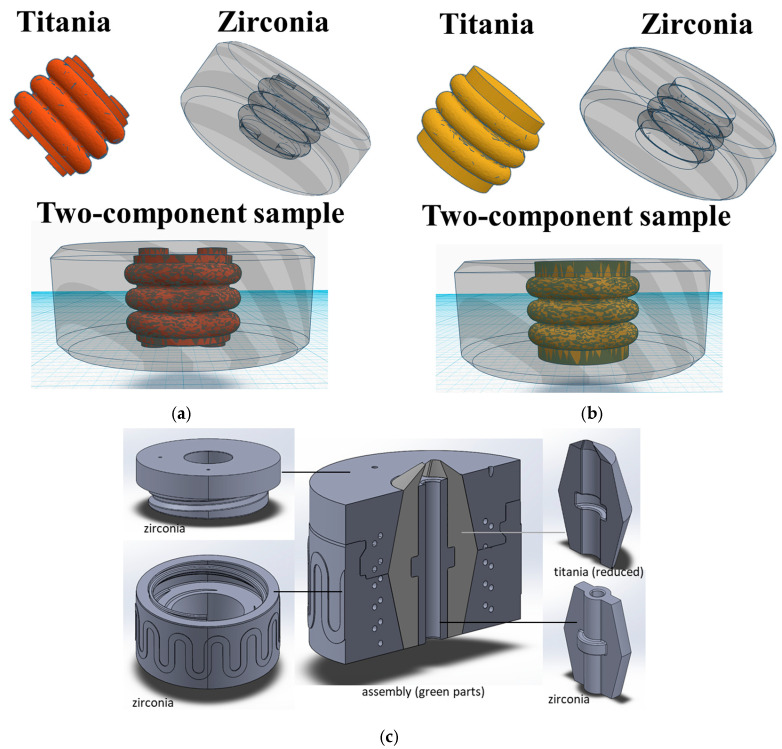
Schematic representation of two-component samples of type 1 (**a**) and type 2 (**b**) printed simultaneously by Multi-CAMP. Design of two-component element assembled after printing and sintered is presented in (**c**).

The single-component samples were investigated in their green state. The two-component samples were simultaneously printed with the Multi-CAMP system. The samples were investigated in the green state and after sintering. The schematic representation of the samples is presented in Figure 2a,b. The last sample consisted of two separately printed single-component ceramic elements. The elements were assembled, sintered, and imaged under OCT as a two-component element. The schematic representation of the sample is presented in Figure 2c.

Optical Coherence Tomography is a volumetric imaging method based on a Michelson interferometer. The first implementation of OCT was presented by Huang et al. in 1991 [24]. Since then, several modified implementations of OCT have been developed. Detailed information on the architecture of the different OCT systems and their application in the field of medicine and biology can be found elsewhere [24,25,26]. This work employs Fourier Domain OCT (FD-OCT) also known as Spectral Domain OCT (SD-OCT) [27]. For the sake of better understanding the results presented here, we will briefly describe the physical foundation of the method as well as the basics of signal processing and data representation.

**Figure 3:**
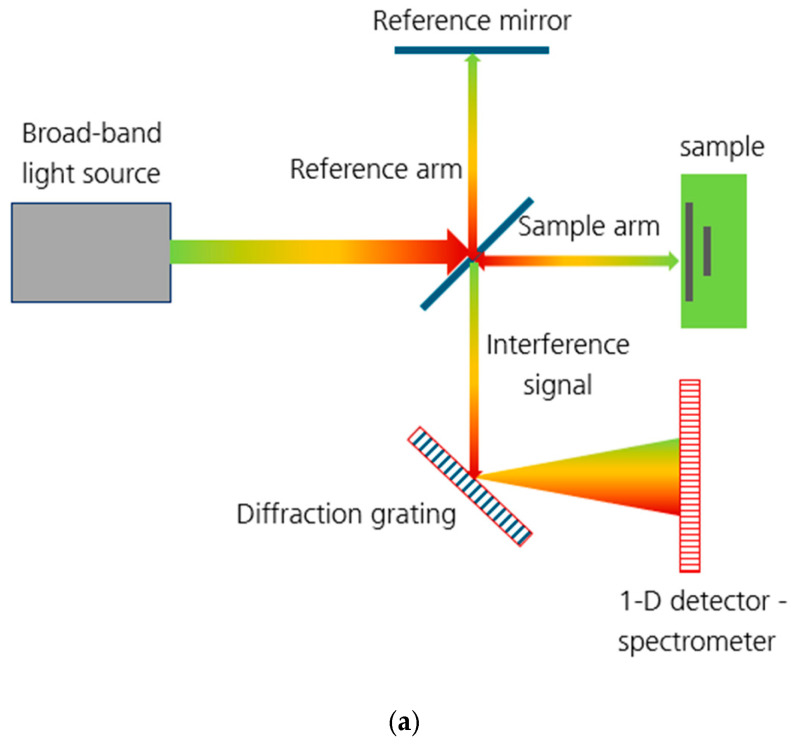

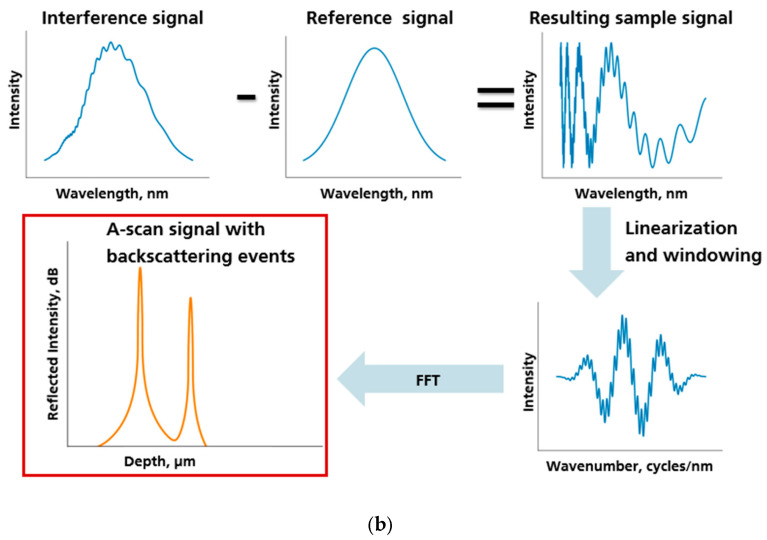
Schematic representation of an SD-OCT system (**a**) and the corresponding chain of signal formation (**b**).

A schematic representation of an SD-OCT system is presented in Figure 3a [28]. A light source (laser or a super-luminescent diode) emits broadband, temporally short-coherent light with wavelengths from the near-infrared part of the electromagnetic spectrum. The light is split into two parts, namely a reference and a sample arm. The reference arm is terminated with a simple flat mirror, which serves as a reference reflector. The sample arm is terminated with an imaging system, often a combination of a 2-axis point-scanning system and telecentric lens, whose focal plane is set to be just below the sample surface. Depending on the transparence of the material, the light can penetrate the subsurface up to several millimeters in depth. It is partially backscattered at the sample surface and at scattering centers in the sample volume. The backscattered light interferes with the reference light and produces oscillations in the intensity as a function of wavelength. The interference signal is dispersed by a diffraction grating and the individual wavelength components are detected by an array detector (spectrometer). The background intensity from the spectral shape of the light source is then subtracted to isolate the interference pattern, which contains information related to the presence of the scattering centers within the sample. To convert it into a depth profile, Fast Fourier Transform is often used, requiring several pre-conditioning operations. The signal from the spectrometer is linear in the wavelength and must first be interpolated so that it instead becomes linear in wavenumber. A windowing function must be applied so that the signal is smooth and continuous at the edges. The so-called zero-padding is often used to smooth the final signal and improve the quality of any peaks present. Only then can the Fast Fourier Transform be applied. The use of a discrete Fourier Transform results in the loss of the sensitivity of the signal as a function of depth due to the finite sampling of the spectrometer [29,30].

**Figure 4:**
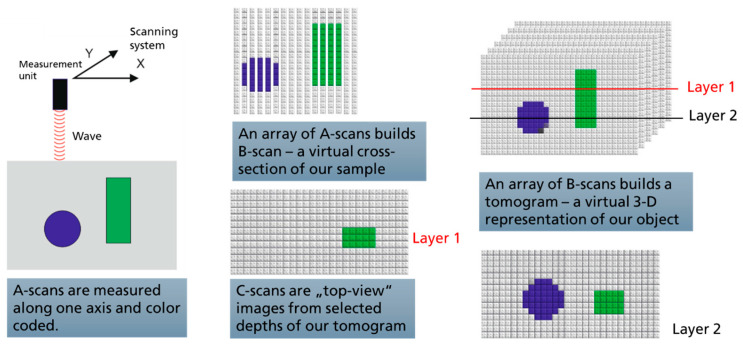
A-scan is the main building bloc of the tomogram in OCT. It is recorded first for scan-wise building the so-called B-scan. A-scans and B-scans can be analyzed in situ. After the tomogram is complete, it can be re-cut in different planes, allowing for the analysis of the top-view images from different depths—the so-called C-scans.

The signal processing chain, presented schematically in Figure 3b, results in the so-called A-scan. The changes in the intensity of the reflected light as a function of depth inform us about the presence of scattering centers within the probed volume. As the laser is moved along the fast scan axis, A-scans are acquired for each of the defined raster points, providing a 2-D reflectivity profile. This is the so-called B-scan, which can be interpreted as a virtual cross-section of the illuminated volume. Consecutive B-scans can be acquired by incrementing the scanning system along the slow scan axis, to produce a 3-D volume of reflectivity within the scanned area—the tomogram. Once the tomogram is recorded, cross-sections parallel to the sample (C-scan) surface can be extracted. The schematics of the tomogram acquisition is presented in Figure 4.

The lateral resolution of an OCT system is determined by the numerical aperture of the lens focusing the sampling light [30,31,32]. According to Abbe’s law:

(1) $$∆x=1.22\frac{\lambda }{2NA}$$

where *λ* is the central wavelength of the source and *NA* is the numerical aperture of the lens. One can increase the lateral resolution of an OCT system by using a lens with higher aperture; however, this comes at the cost of a reduced depth of focus in *Z,* which is given as:

(2) $${DOF}_{Z}=2\frac{\lambda n}{{NA}^{2}}$$

where *n* is the refraction index of the sample. Therefore, one must always balance the lateral resolution against the depth range of OCT. For example, in the case of optically transparent materials, the use of a lens with high numerical aperture to chase a high lateral resolution might overly restrict the depth of field to a much smaller fraction of the sample than the rest of the system would support.

The axial (or depth) resolution of OCT systems depends only on the temporal coherence length of the light source. For light sources with Gaussian intensity distribution, the coherence length can be defined as:

(3) $$l_{c}\approx \frac{0.44}{n}\frac{\lambda_{0}^{2}}{\Delta \lambda }$$

As can be seen in Equation (3), one can improve the lateral resolution of the OCT system by reducing the center wavelength or increasing the bandwidth of the source. In addition, the axial resolution may vary for different materials, depending on their refraction index [26].

The usable volume in an OCT tomogram depends on several factors, such as the transparency of the object at the given wavelength, number of scattering elements within, and how large the depth of focus of the system is. Most of the commercially available systems use wavelengths of about 900 nm and 1300 nm and bandwidths up to 100 nm. The choice was justified by the historical development of the systems for the investigation of biological tissue and the availability of optical components required for the construction of such systems. When applied to ceramic materials, the performance of the OCT system varies, as “ceramics” is a broad term that encompasses many materials with many varying properties. With the system used in this work, one can obtain visual information up to a 4 mm depth for optically transparent ceramics. In the case of strongly backscattering ceramics, called here turbid ceramics, the penetration depths do not exceed 300 µm. For opaque ceramics, such as sintered titanium oxides or carbides, depth information is not available, and OCT can only provide the information on the sample topography.

The system used in this study was a commercially available Thorlabs Telesto II system, which operates at a 1325 nm central wavelength. The rate of A-scan acquisition was 76 kHz. No averaging was performed during the measurements. The scan sizes varied depending on the size of the sample and the size of the selected region of interest. The lateral pixel size was 10 µm, unless otherwise stated. All the measurements reported here were obtained ex situ.

## 3. Results and Analysis

### 3.1. Qualitative Analysis at B-Scans Level

**Figure 5:**
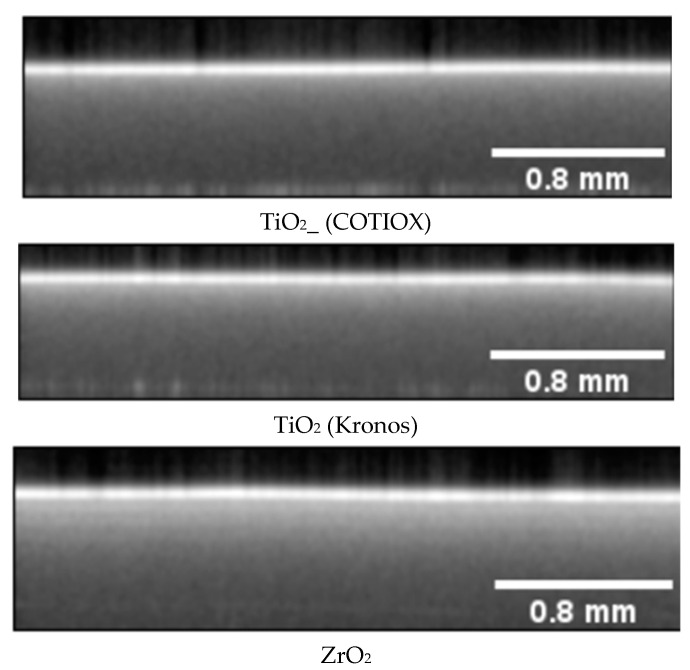
Mean intensity B-scan calculated from 10 subsequent B-scans selected at random positions in the tomogram. Visual inspection does not reveal any differences in the images except for small horizontal variations in the scan obtained on the ZrO2 sample.

Typical OCT results are presented in the form of either B-scans or C-scans. These are selected cross-sections or top views from a selected depth level, respectively. Both views contain valuable information about the sample’s homogeneity. Figure 5 presents examples of typical B-scans recorded on green samples of ZrO_2_ and TiO_2_ printed by the Lithoz printer.

At a first glance, the three B-scans look identical. There appears to be no difference between the images acquired on the two TiO_2_ samples. There are faint horizontal lines visible in the ZrO_2_ sample. The expected thickness of the printed layer was about 25 µm. Thus, with help of the five lines visible in the B-scan obtained on the ZrO_2_ sample, we can estimate the penetration depth to about 120 µm. We use the term estimated, as precise determination is limited by the assumption of the refractive index of the printed ZrO_2_ in the green state and the relatively large axial pixel size (7 µm in air). We could not see any significant variations within the individual samples, which indicated that samples were very homogenous and free of buried defects.

**Figure 6:**
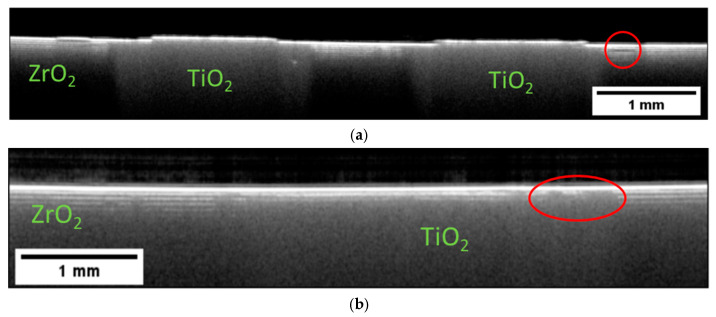
Intensity-averaged B-scans calculated from subsequent B-scans measured in the relevant regions on the Multi-CAMP samples of (**a**) type 1 and (**b**) type 2. The samples were in the green state.

OCT tomograms were also obtained on the Multi-CAMP samples type 1 and 2. Selected examples of B-scans are presented in Figure 6 for green samples of type 1 and 2. In image (a) we can distinguish differences in the regions consisting of ZrO_2_ (matrix) and the two circular structures of TiO_2_. The main difference is the pronounced layered character of the ZrO_2_ in contrast to the homogenous appearance of TiO_2_. Small variations in the ZrO_2_ region are easily visible as they affect the continuity of the printed layers. In some areas the line contrast appears to be stronger. This means that the light is backscattered to a larger extent in those areas, indicating larger differences in the optical properties at the interface between two subsequent layers. Such variations in the amplitude of the optical signal along one horizontal line may indicate process variations during the application of the next layer.

**Figure 7:**
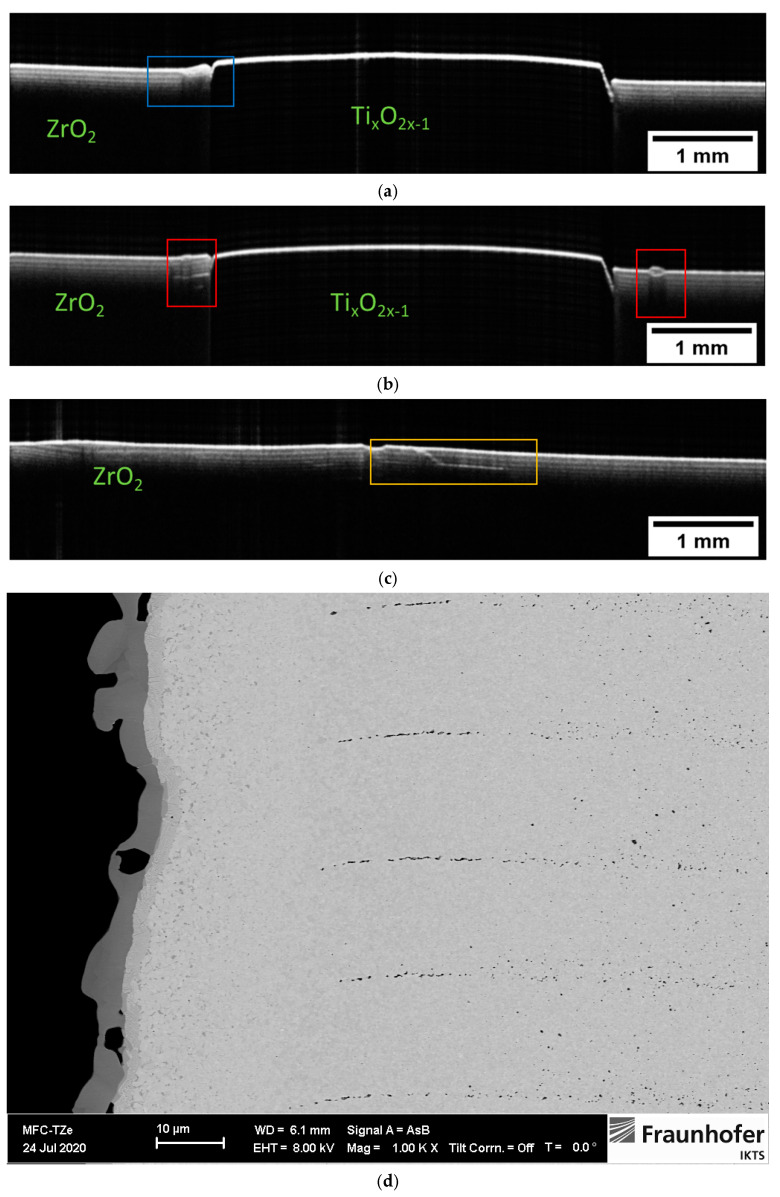
Intensity-averaged B-scans calculated from subsequent B-scans measured in relevant regions on the sintered two-component sample assembled after printing single-component elements are shown in in (**a**–**c**). SEM image presented in (**d**) shows the presence of pores forming between the layers during the sintering. Their presence is detected by backscattered light and visualized as layers in the OCT images. The thickness of the sintered layer is about 16 µm.

Sintering is the obligatory processing step that enables the densification and phase transformation necessary for the ceramic to gain the desired properties. Due to the high temperatures and often significant material shrinkage, there is a high chance of damage during the process. It was therefore essential to acquire OCT tomograms on sintered samples as well. Figure 7 presents B-scans extracted from a two-component structure, which was assembled from green single-component ceramic elements and then sintered. In each element, the ceramic content and size were selected to account for the different shrinkage of the parts during sintering to ensure their shape conformity after sintering.

Let us compare the B-scans presented in Figure 5 and Figure 7. It can be easily seen that the sintering process changed the effective optical properties of the two materials. The TiO_2_ now transformed into Ti_x_O_2x−1_ became opaque and the optical signal is already strongly backscattered at the surface. The pronounced changed in the optical properties of Ti_X_O_2x−1_ is caused by a loss of lattice oxygen [33,34]. The ZrO_2_ region, on the other hand, became more transparent due to increased density. The layered character of this region is now more pronounced. One can easily count at least four layers. The reason for the enhanced visibility of the ZrO_2_ layers is the presence of small pores forming between the layers during the sintering. As the pores cause the light to backscatter, the layer interface appears as a separate structure. The layers are consistently visible within the whole imaged area, indicating a consistent presence of pores. An SEM image validating this explanation for the contrast of the OCT images is shown in (d). The SEM image also provides the opportunity to estimate the penetration depth of OCT for this 3-D-printed material after sintering. As mentioned in previous sections, the thickness of the layer in the green state was about 25 µm. The SEM image shows that the layer thickness shrank down to about 16 µm after the sintering. As we can distinguish about six to eight layers in the corresponding B-scans shown in Figure 7a–c, the estimated penetration depth for this OCT system was about 130 µm.

The predominantly homogenous appearance of the layers makes it easy to identify variations. For example, in Figure 7a, one can see a small deformation and lack of a layered structure in a small region on the left side of the Ti_x_O_2x−1_ core. In Figure 7b, we can see the small but continuous deformation of the first several layers in direct proximity to the Ti_x_O_2x−1_ core (area marked on the left). The brighter stripe visible within the volume may indicate the presence of a small crack. In the same figure, we can see a small deformation of the surface, obscuring the view into the sample volume. Figure 7c presents a B-scan obtained within the ZrO_2_ region only. One can see deformations of the ZrO_2_ layers, especially on the left side of the B-scan. In the middle of the image, we can see a distinct feature that could be interpreted as a fracture that extends across multiple layers to the sample surface.

**Figure 8:**
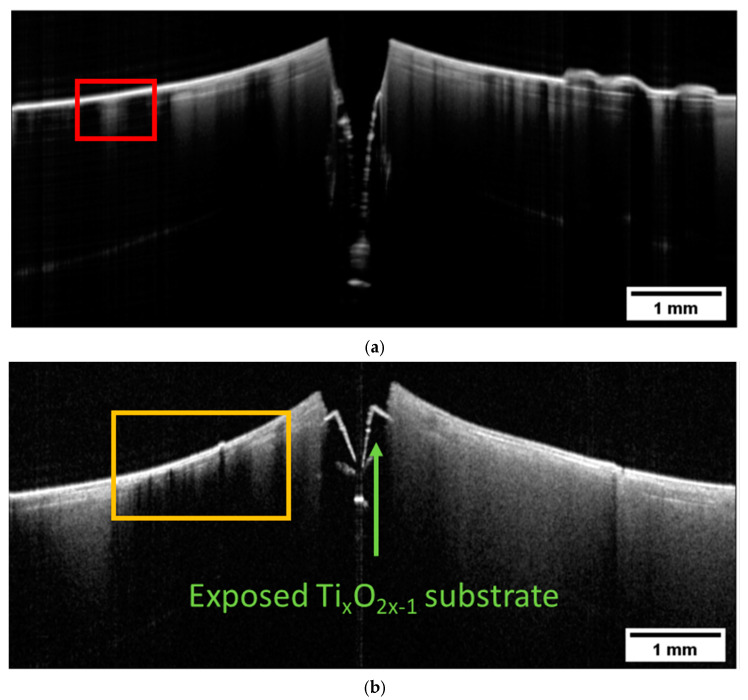

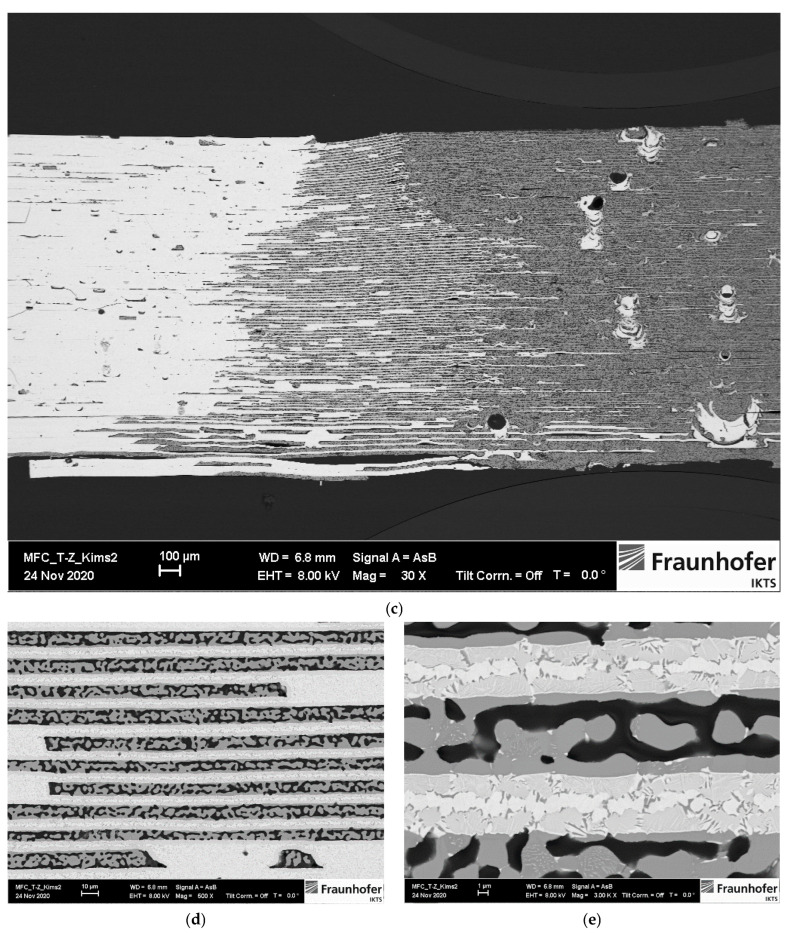
Intensity-averaged B-scans calculated from subsequent B-scans measured in relevant regions on the sintered Multi-CAMP sample of type 1 in (**a**,**b**). SEM images in (**c**–**e**) show the mutual contamination within the phase boundary. ZrO_2_ and Ti_x_O_2x−1_ are overlapping. Large pores can also be detected.

B-scans were also measured on Multi-CAMP samples. Selected examples of B-scans measured on sample type 1 are presented in Figure 8. They show the cross-sections of a deformed sample with an obvious fracture caused by the premeditated defects introduced to the samples during the printing process. This result shows what happens when the difference in the thermal expansion coefficients of the two components and the complex architecture of the structure is not accounted for. The layer of the ZrO_2_ peeled off and exposed the underlying Ti_x_O_2x−1_. Again, the TiO_2_ becomes opaque after the sintering due to the loss of lattice oxygen, whereas the ZrO_2_ becomes more transparent due to the increased density. However, the variations in the ZrO_2_ are very complex. In some parts, the ZrO_2_ appears to be transparent (the left side of the Figure 8a), while in others, the sample is more turbid with visible layers. Even though the B-scans were obtained on the same sample, the appearance of the ZrO_2_ in image (a) differs significantly from that in image (b).

The contrast of the OCT images presented in Figure 8 could be explained with the help of SEM images. The SEM image in Figure 8c shows that there is a cross-contamination between the two materials. Titania and zirconia are overlapping at the interface. As this contamination was present already during the green state, we can understand now why we were able to see a clear layered structure in the two-component samples. The sintering process only enhanced the optical differences between the two materials, which is also reflected in the pronounced structures visualized by OCT. The presence of pores, an additional optical factor, can also be observed. The conclusion is clear—simultaneous printing of materials in Multi-CAMP technology requires careful washing. The ability of OCT to detect cross-contamination was one of the most important criteria for performance evaluation for its future implementation as an in-line monitoring technique.

**Figure 9:**
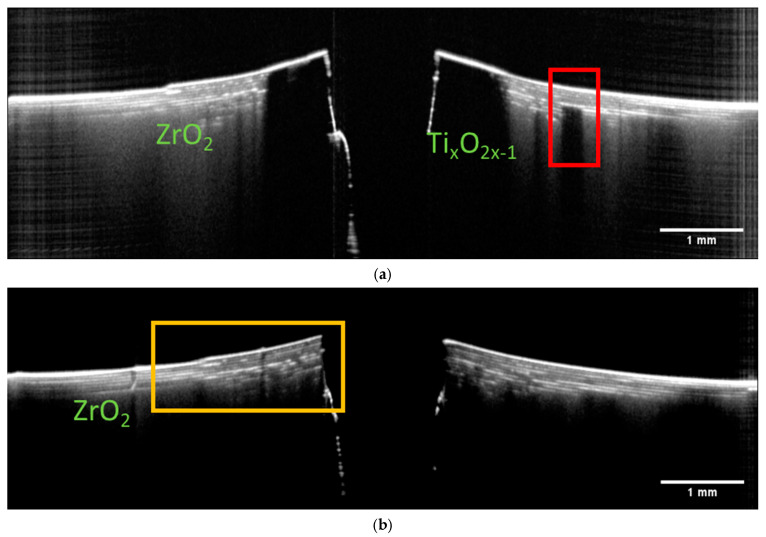
Intensity-averaged B-scans calculated from subsequent B-scans measured in selected relevant positions (**a**,**b**) on the sintered two-component sample of type 2 printed with a custom-designed prototype printer at KIMS.

To finish the overview, in Figure 9 we present the B-scans obtained on the two-component sample of type 2. Again, the sample is fractured, and the surface is strongly deformed. There is a clear distinction between the opaque Ti_x_O_2x−1_ and ZrO_2_. In this case, we can see variations in the optical properties of the ZrO_2_ as a function of depth. We have regions with more translucent ZrO_2_ followed by regions where light was strongly backscattered. Even opaque structures can be distinguished (right side in Figure 8a). The layered structure of ZrO_2_ is strongly pronounced.

The results presented above clearly show that OCT has the potential to detect surface and buried defects in 3-D-printed ceramics in the green and sintered states. In the case of opaque ceramics, such as Ti_x_O_2x−1_, one can extract information on the position of the sample surface and thus on the topography and geometry of the sample. In the case of turbid and translucent ceramics, the penetration depths extend from a few hundreds of micrometers to several millimeters. As was shown in the paper by Zorin et al., it is possible to construct an OCT system with a mid-infrared light source. The use of much longer wavelengths enables the system to obtain information from depths exceeding 500 µm [23]. The ceramics investigated in the cited study were alumina and zirconia ceramics in green state. Reference images obtained with a commercially available Thorlabs system operating at 1300 nm were obtained to highlight the improved performance of the mid-infrared system. The data acquisition rates of the mid-infrared system are currently significantly lower than those of the near-infrared systems, but the images presented here show that the OCT methods have untapped potential as in-line monitoring systems. Zorin’s paper concentrates mostly on the improved performance of the mid-infrared system in terms of penetration depth and says very little about a possible approach to data reduction and defect detection. OCT is a tomography method and delivers large amounts of data. In the most traditional approach, B-scans or C-scans are analyzed with image processing and pattern recognition. Those approaches can be very effective [35] but are often time consuming both in preparation and in-line performance. A large collection of reference images is required for comparison and verification. Image processing means waiting with the analysis until after a full B-scan or tomogram (for C-scan processing) is acquired. On top of that, in the case of turbid ceramics, the resulting signal-to-noise ratio is relatively low, and the signal intensity changes strongly as a function of depth. This means that simple tools for defect detection such as thresholding will work only on a small part of the image, if at all. Therefore, we propose another solution. Instead of waiting for full images to be acquired and analyzing them one after another, we looked directly at the A-scans.

### 3.2. Data Analysis at A-Scan Level

**Figure 10:**
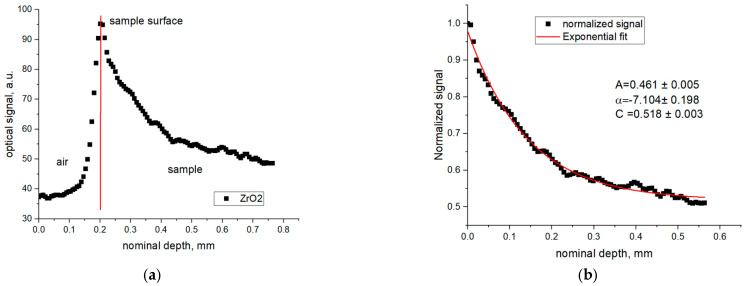
Example of a typical A-scan recorded on green 3 D-printed ZrO_2_ sample in (**a**). Surface of the sample is represented by the signal maximum. The optical signal decays exponentially within the sample because of dominant scattering within the sample. The signal was normalized to the maximum value and the resulting data (**b**) were fit with an exponential decay curve.

An A-scan is a one-dimensional array of data. An example of an A-scan acquired on a ZrO_2_ sample is shown in Figure 10. Such a signal is typical for turbid ceramic materials. The signal backscattered at the surface is the strongest. One can easily detect the position of the maximum and use it to reconstruct surface topography. Additionally, one can extract the maximum value and use it as an imaging quantity to show how differently the light is backscattered from different locations on the surface. Those differences stem from changes in the surface topography as well as reflectivity.

As the light enters the sample, we can see that the signal decays exponentially. It fits well with our expectations for strongly scattering mediums. It was shown in [36] that the simplified attenuation law can be used to describe the decay of the OCT signal in turbid media for shallow imaging depths. Levitz et. al, proposed a more detailed equation including the components responsible for multiple scattering in [37]. Those and similar approaches have been used to determine attenuation coefficients on various tumorous and healthy tissues [38,39,40].

We used a simplified form of an equation proposed by Levitz [37] to describe the decay of the signal in the 3-D-printed ceramics.

(4) $$I\left(z\right)=Ae^{\alpha z}+C$$

*I*(*z*) is the signal intensity as measured by the OCT system, *z* is the depth, and *A* is a constant combining the system parameters (e.g., source intensity and optic path attenuation, which are assumed constant across measurements) and backscattering coefficient of the sample. *α* is the decay parameter describing how fast the signal is attenuated within the sample. *C* in this case is an offset accounting for the multiple scattering as well as for the mixed component of the single and multiple scattering.

We do not attempt to determine the quantitative physical value of the attenuation coefficient of the 3-D-printed ceramics. The presence of a polymer binder, possible variations in ceramic powder grain size and quality, as well as density variations in the final mixture are only a few factors influencing the complexity of the material. In addition, the green state of the material is temporary and is modified in the sintering process. As such, the refraction index of such a 3-D-printed sample is unknown and likely nonuniform; the resulting uncertainty in the depth calibration will be difficult to determine. Therefore, there is really no need, from the practical point of view, to construct a complex model with ill-defined parameters. Instead, we attempted to determine a scalar, which we refer to as the decay parameter. Its role is to tell us about the relative changes within the sample and help us to identify local variations, while reducing the overall amount of data. A similar approach to the analysis of data obtained on ceramic materials was proposed by Toyokura [41]. In his study, he integrated a selected part of the A-scan and used the value to illustrate density variations in a green alumina sample.

Figure 10 presents a typical A-scan recorded on a green 3-D-printed ZrO_2_ sample. The surface of the sample is easily identified as it backscatters the incident light strongly. The light intensity at the surface is determined and used to normalize the remaining part of the A-scan. The normalized signal is fit with an exponential decay curve. The fit parameters, namely amplitude and the decay parameter, are used as separate imaging quantities. In addition, we display the maximum value as a 2-D image showing how the surface reflects the light.

**Figure 11:**
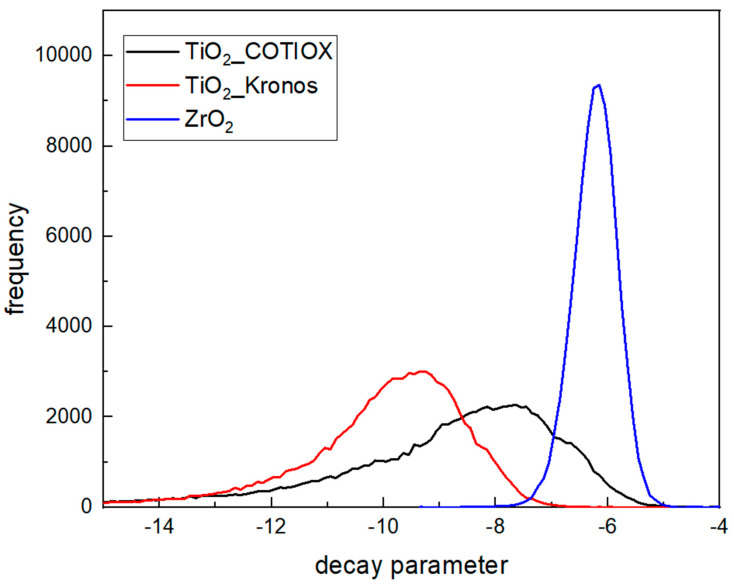
Distribution of decay parameter determined for A-scans measured on 3-D-printed green samples of TiO_2__COTIOX, TiO_2__Kronos, and ZrO_2_.

Decay parameter values were determined first for the blank samples (see Table 1 and Figure 4). The A-scans were normalized to the maximum value of the signal and fit with the exponential decay function as described above. The determined values for the decay parameter from the whole tomogram were analyzed in terms of the range and the distribution of the values. The histograms determined for the three blank samples are presented in Figure 11.

As can be seen in the figure, there is a difference in the values of the decay parameter determined for the two TiO_2_ samples and the ZrO_2_ sample. The distribution determined for the ZrO_2_ sample is much narrower than those obtained for TiO_2_, indicating the uniform grain size of the ceramic powder and very homogenous character of the printed sample. The distributions determined for the TiO_2_ samples are much broader in comparison. Furthermore, there are differences between the two TiO_2_ samples, which may be attributed to the use of powders from different vendors. The differences in the distribution of the decay parameter could be explained by differences in the mean size of the grains. According to the vendors, the mean grain size was 0.4 µm and 0.3 µm for the Kronos and COTIOX powders, respectively. In addition, phase composition, namely the amount of rutile, is higher in the small-grained powder. For comparison, the mean grain size of the zirconia powder was 0.7 µm. The optical response of the samples seems to correlate with the differences in the mean grain size of the powders. However, these are preliminary results and additional studies should follow to separate complex interaction of various factors such as: effective refraction index of the precursor powder and binder, mean grain size of the precursor, and imaging parameters such as focus position, settings of the reference arm, etc.

**Figure 12:**
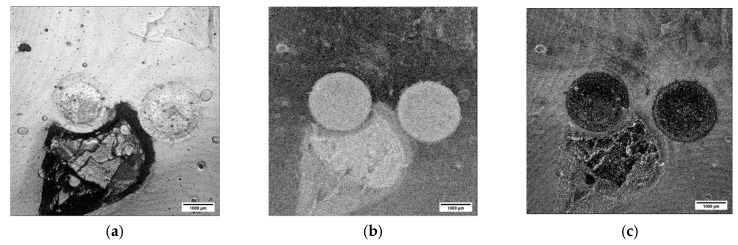
A-scans obtained on a 3-D-printed two-component sample of type 1 were analyzed and the following parameters were extracted and used as imaging quantity: (**a**) light intensity at the surface, (**b**) decay parameter, and (**c**) amplitude of the exponential fit.

The results presented in Figure 11 indicate that the variations in the decay parameter could be used for the visualization of material variations or inhomogeneities within the material itself. To test this hypothesis, the decay parameter was determined from tomograms acquired on a 3-D-printed two-component sample (type 1) in the green state (see Figure 5 and Table 1). In addition, we displayed the intensity values determined at the sample surface and the corresponding amplitude of the exponential fit. The resulting images are presented in Figure 12.

Figure 12a presents the light intensity at the surface. The gray values of this image are influenced by the local reflectivity and topography of the sample. The darker region in the lower part of image (a) was correlating with the distinct surface damage that occurred during shipment. The irregular surface features resulted in the reflected light not reaching the detector, leading those parts of the sample surface to appear much darker than the rest. The decay parameter image is shown in (b). One can easily identify the circular titania structures. In addition, one can recognize that part of the buried titania was exposed in the damaged area. Part of the arch of the titania base can be recognized in the lower left side of the structure (please see Figure 1 for reference). As indicated by the decay parameter image, the ZrO_2_ matrix appears relatively uniform across the imaged area. Within the damaged areas showing the largest topography variations, the decay parameter has consistent values which differ from both titania and zirconia regions. The fit amplitude image is shown in (c), where one can also easily differentiate between the two material phases.

**Figure 13:**
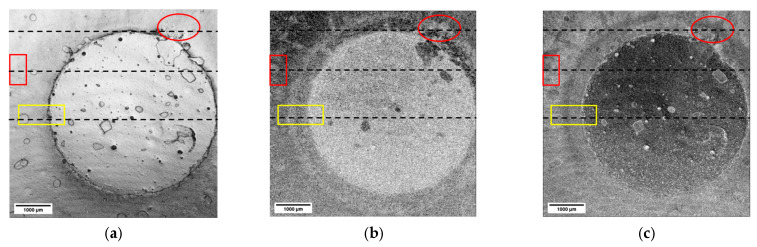
A-scans obtained on a 3-D-printed two-component sample of type 1 were analyzed and the following parameters were extracted and used as imaging quantities: (**a**) light intensity at the surface, (**b**) decay parameter, and (**c**) amplitude of the exponential decay. Decay parameter and amplitude images showed additional features not visible in the surface intensity image. Those were marked, and corresponding B-scans were extracted for further analysis.

**Figure 14:**
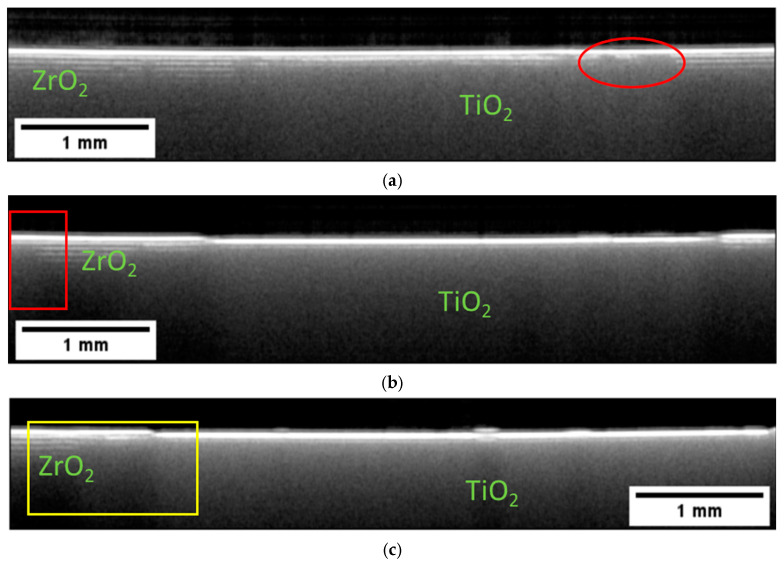
Subsequent B-scans were intensity-averaged. They were selected from regions marked in Figure 13 with the help of a (**a**) red oval, (**b**) red rectangle and (**c**) yellow rectangle. Features visible in the B-scans correlate very well with the corresponding variations in the values in the decay parameter.

A similar analysis was conducted on a two-component sample of type 2. The images constructed from the values of the light intensity at the sample surface, decay parameter, and fit amplitude of the exponential decay are presented in Figure 13. The surface intensity image (a) shows the central round core as the printing process influences the sample topography. Additional features are visible in images of the decay parameter (b) and fit amplitude (c) that have no corresponding equivalent in the surface intensity image. Selected areas of interest are marked in yellow and red. B-scans were extracted from the regions marked and are shown in Figure 14 below.

Coordinates of the defects as identified in the decay parameter image were used to locate the relevant B-scans and the position of the defects within the B-scans. We selected several neighboring B-scans from the relevant regions and calculated intensity-averaged B-scans. The number of the subsequent B-scans used for averaging ranged from five to ten, depending on the size of the defects. The regions of interest from Figure 12b are marked equivalently in the corresponding B-scans. It is immediately visible that the selected regions differ in some way from the surrounding area. For example, the region marked in Figure 13a is missing reflections at the layer interfaces, which are present throughout the rest of the B-scan. At this point we can only speculate about the factor responsible. It could be local variation in the quality of the print, density variation, contamination, or others. A separate SEM/FIB study must be performed at such a location to identify the cause. At this point, we are satisfied with the ability to identify the variation in the print quality. The same phenomenon is observed in Figure 13b.

Figure 13b clearly shows the presence of a ring around the TiO_2_ core. The corresponding B-scan shown in Figure 14c shows that the appearance of the layers also varies in this region. In this case, we presume that there is an interface formed where ZrO_2_ is contaminated with TiO_2_.

**Figure 15:**
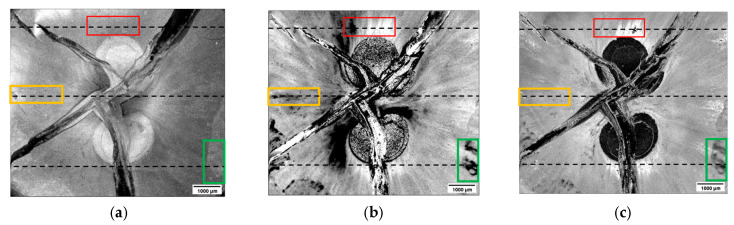
AA-scans obtained on a 3-D-printed and sintered two-component sample of type 1 were analyzed, and the following parameters were extracted and used as imaging quantities: (**a**) light intensity at the surface, (**b**) decay parameter, and (**c**) amplitude of the exponential decay fit. Decay parameter and fit amplitude images showed additional features not visible in the surface intensity image. Those were marked by rectangles of different colors, and corresponding B-scans were extracted for further analysis.

The sintering process changes the optical properties of the ZrO_2_ and TiO_2_. The changes were clearly visible with our processing method, as shown in Figure 7, Figure 8 and Figure 9. The differentiation between the Ti_x_O_2x−1_ and ZrO_2_ was strongly pronounced, as Ti_x_O_2x−1_ becomes opaque, exhibiting an absorption band that extends from visible light to near-infrared wavelengths [34]; ZrO_2_ becomes more transparent in comparison to its green state. Accordingly, the decay characteristic of the corresponding A-scans changed as well. The values of the light intensity at the surface, decay parameter, and the amplitude of the exponential fit were determined from a tomogram obtained on sintered two-component samples of type 1 and is presented in Figure 15.

**Figure 16:**
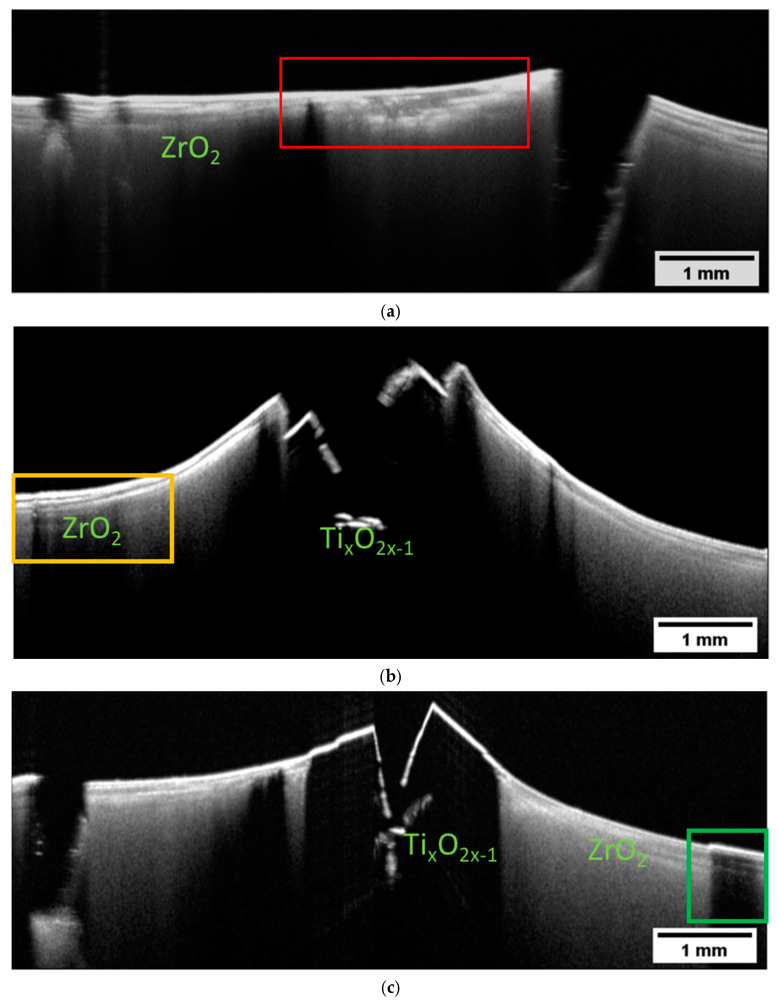
Subsequent B-scans were intensity-averaged. They were selected from regions marked in Figure 15 with (**a**) red (**b**) yellow, and (**c**) green rectangles. Features visible in the B-scans correlate well with the corresponding variations in the values in the decay parameter.

All the three images in Figure 15 show the complex fracture. Small variations in the light intensity at the surface are also visible. However, those variations are not as strongly pronounced as those visible in the decay parameter image shown in Figure 15b. The two Ti_x_O_2x−1_ structures appear relatively uniform; however, strong variations are visible within the ZrO_2_ region. Three exemplary areas were selected and marked with differently colored rectangles. There, the corresponding B-scans were isolated and intensity-averaged. The results are presented in Figure 16.

The results shown in Figure 14 and Figure 16 show that there is a correlation between the variations of the decay parameter values and the variations in the optical properties of the material. Those variations can be material differences, as well as density variations, contamination, or delamination. As the origin of those variations is related to the complexity of the material, the 3-D printing process, and material interactions during the printing process, it is not easy to interpret the OCT images. Additional studies combining the OCT and SEM methods can help to interpret such data in the future.

## 4. Summary and Outlook

We used OCT to obtain volumetric information from 3-D-printed ceramic structures in green and sintered states. The selected initial materials were ZrO_2_ and TiO_2_ and they were used to print single- and two-component structures. The OCT tomograms obtained for the green samples showed that both ZrO_2_ and TiO_2_ are optically turbid materials, and the measured optical signal displays strong exponential decay character. The penetration depths were up to 130 µm, which allowed for the investigation of several printed layers at once. OCT images obtained on sintered samples showed the influence of the sintering on the optical properties of the materials tested and the evolution of defects in the optically translucent ZrO_2_.

Using the B-scans to visualize the presence of buried defects or material variations belongs to standard tomography approaches. The B-scans showed variations in the intensity values stemming from layer interfaces, defects, and material variations. Already, this simple analysis showed that OCT can be used to detect surface and subsurface defects in the 3-D-printed ceramic structures. However, automized analysis of OCT B-scans can be challenging due to low SNR, strong signal decay as a function of depth, and the complex character of the signal variation. Therefore, the idea of analysis at the level of A-scans offers better efficiency. The proposed procedure to identify the surface position and fit an exponential decay function to the 1-D A-scan with a predefined range is fast and can be performed in real time, inline to the data acquisition without buffering. The output of the analysis is reduced to several parameters, which reduces the volume of the transferred/stored data significantly. The information obtained provides feedback on the surface quality and material variation within the imaged volume. Data points laying outside of the expected range during in-line monitoring can be used as signals for the presence of defects for further inspection.

In this paper, we focused on the detection of buried defects via the analysis of A-scans and showed the use of the obtained fit parameters. Although the extraction of the topography of the printed structures worked well, it was omitted for conciseness and will be included in future works. The combined capability of OCT to characterize the surface of the printed objects and to detect defects buried below the surface makes OCT a perfect candidate as a quality control tool for 3-D-printed ceramic materials.

## Figures and Tables

**Table 1:** Overview of the 3-D ceramic samples investigated with OCT system.

	Green	Sintered
Single-component samples	TiO_2_ (Kronos), Lithoz printer [14] TiO_2_ (COTIOX), Lithoz printer ZrO_2_, Lithoz printer [14]	- - -
Two-component samples	TiO_2_/ZrO_2_ type 1, Multi-CAMP TiO_2_/ZrO_2_ type 2, Multi-CAMP	TiO_2_/ZrO_2_ type 1, Multi-CAMP TiO_2_/ZrO_2_ type 2, Multi-CAMP TiO_2_/ZrO_2_ sintered after assembling single-component elements, Lithoz printer

## Data Availability

Not applicable.
